# Nasal microbiota predictors for methicillin resistant *Staphylococcus* colonization in critically ill children

**DOI:** 10.1371/journal.pone.0316460

**Published:** 2025-01-15

**Authors:** Kathleen Zani, Joseph Hobeika, Yilun Sun, Christina Kohler, Anju Cherian, Trinity Fields, Qidong Jia, Li Tang, Nicholas D. Hysmith, Elisa B. Margolis

**Affiliations:** 1 Department of Pediatrics, Division of Critical Care, University of Tennessee Health Science Center, Memphis, TN, United States of America; 2 Department of Infectious Diseases, St Jude Children’s Research Hospital, Memphis, TN, United States of America; 3 Department of Biostatistics, St Jude Children’s Research Hospital, Memphis, TN, United States of America; 4 Department of Pediatrics, Division of Infectious Diseases, University of Tennessee Health Science Center, Memphis, TN, United States of America; University of Calgary, CANADA

## Abstract

**Background:**

Surveillance cultures to identify patients colonized with methicillin-resistant *Staphylococcus aureus* (MRSA) is recommended at pediatric intensive care unit (PICU) admission but doesn’t capture other methicillin-resistant *Staphylococcus* and is resource intensive. We determined the prevalence and identified nasal microbiome predictors for methicillin-resistant *Staphylococcus* colonization at the time of PICU admission.

**Study design:**

A prospective cohort study was performed in a 20-bed pediatric intensive care unit (PICU) between 2020–2021. Anterior nares nasal swabs processed for MRSA culture, nasal microbiome and *mecA+* qPCR were obtained within first five days after PICU admission. Predictive values of methicillin-resistant *Staphylococcus* carriage on symptoms of infection and for nasal microbiome attributes were calculated.

**Results:**

A total of 5 (8.0%) of 62 patients had a nares culture positive for MRSA and 22 (35.5%) of 63 patients had methicillin-resistant *Staphylococcus* (MRSA or methicillin-resistant coagulase-negative *Staphylococci*). In univariate analysis, carriage with MRSA or MRCoNS was associated with having a fever during PICU stay. Colonization with a distinct set of microbes (including *Haemophilus*, *Streptococcus*, *Prevotella* and *Corynebacterium* sp.) was predictive of having methicillin-resistant *Staphylococcus* colonization.

**Conclusions:**

Carriage with methicillin-resistant *Staphylococcus* may lead to transmission in critically ill pediatric patients. Carriage of particular nasal microbes appears to facilitate colonization with methicillin-resistant *Staphylococcus*.

## Introduction

*Staphylococcus aureus* is a leading pathogen causing hospital-acquired infections in children [1]. Approximately five percent of children admitted to pediatric intensive care units (PICUs) are colonized with methicillin-resistant *Staphylococcus aureus* (MRSA) [2]. Studies have proven that MRSA colonization increases the risk of subsequent invasive MRSA infection in both children [3] and adults [4–6]. Children who acquire MRSA after admission to the PICU are at a higher risk for invasive disease [3] and detection via PCR is sensitive to predict MRSA infection [7]. Colonization pressure within a critical care area is directly related to MRSA transmission in children. Popoola et al. showed that colonization pressure above 10% increases transmission of MRSA by 3-fold [8], leading to widespread screening and decolonization programs.

Coagulase-negative *Staphylococci* (CoNS), once thought to be largely commensal organisms, are now increasingly recognized as significant healthcare-associated pathogens, especially in patients with indwelling medical devices common in PICUs [9]. The rising prevalence of methicillin resistance among CoNS strains, which can be as high as 80%, limits the effectiveness of first-line antibiotics and complicates treatments [9]. This resistance contributes to increased morbidity, mortality, and healthcare costs [9], although CoNS has not yet been included in widespread screening programs. To understand the value of PICU infection control strategies, this study includes screening for both MRSA and methicillin resistant CoNS.

If the aim of PICU screening is to decrease transmission of hard-to-treat *Staphylococcus* within critical care area, then we hypothesize that there are two key components: a) determining if the prevalence of all methicillin-resistant *Staphylococcus* colonization pressure is high enough for transmission, and b) identifying individuals who may be at higher risk of colonization if exposed due to a lack of colonization resistance in their nasal microbiome. Risk factors for colonization include increased likelihood of exposures (contact with healthcare setting, high endemicity community) [10, 11], selective pressure to maintain (antibiotic use) [12], and colonization resistance from other microbes within the nasal community [13]. While exposure plays a major role in determining who will be colonized by multi-drug resistant organisms, host susceptibility to these organisms may be determined by other flora at the sites of colonization.

Colonization resistance, where the resident microbial community block an intruder’s establishment, plays a crucial role in nasal *Staphylococcus* ecology. The host’s microbial community can prevent colonization by outcompeting the intruder for resources and space; or through active mechanisms like producing antimicrobial compounds, or co-opting host immune responses. The interactions can become sophisticated evolutionary arms races, as seen when S. aureus develops catalase to neutralize H_2_O_2_ produced by *S*. *pneumoniae* or when *S*. *epidermidis* releases serine proteases to inhibit *S*. *aureus* [14]. While these specific antagonistic relationships are well-documented [15] the complex interactions that determine successful colonization remain incompletely understood. Understanding these mechanistic interactions between microbiota and methicillin-resistant *Staphylococcus* is essential for predicting colonization risk. Therefore, this study aims to investigate the prevalence of methicillin resistant *Staphylococcus* in the PICU of a high endemicity community and determine if nasal microbiome signatures can identify patients susceptible to transmission.

## Methods

### Study population and data collection

The study was conducted at the 20-bed PICU of Le Bonheur Children’s Hospital (LBCH), a 255-bed public inner-city pediatric hospital in Memphis, Tennessee from January 1, 2021 through December 31, 2022. The study was approved by the University of Tennessee Health Science Center (UTHSC) Institutional Review Board. Written informed consent was obtained from a parent or legal guardian of all participants, and written assent was obtained from the participant if 8 years or older at the time of approach. Specimens from the anterior nares of all patients that met study inclusion criteria and were consented were obtained for culture and used for *mecA* quantification and nasal microbiome studies. Exclusion criteria included less than one month of age or greater than 18 years, non-English speaking, positive for SARS-CoV-2 virus, or facial trauma or recent surgical procedure that would preclude collection of a nasal swab. Inclusion criteria included patients with concern for infection or sepsis (identified within 5 days of admission to PICU), or patients admitted for post-operative observation.

Data on demographic characterizations, signs indicative of infection, presence of indwelling devices (e.g. central lines, tracheostomies, and ventricular shunts), presence of viral coinfection, history of asthma, and antibiotics usage prior to PICU admission were obtained from hospital records or directly from patients.

### Nares samples processing

Samples were obtained by swabbing the bilateral anterior nares. Cultures were screened on mannitol salt plates with yellow colonies tested for golden hemolytic cultures and oxacillin susceptibility in accordance with NCCLS guidelines. DNA was extracted from nasal swabs using method validated for low bacterial content samples [16]. To determine *mecA* gene presence, quantitative PCR (qPCR) was performed on *mecA* to identify resistance and SCC*mec*-*orfX* junction to identify resistance in *S*. *aureus* genomes [17, 18]. Concordance to culture results were assessed (S1 Table). For nasal microbiome community discernment, the V3-V4 region of 16S rRNA [19] was amplified by Touchdown PCR method [20] (prior to sequencing (Illumina MiSeq paired end 2 x 300bp) at the St. Jude Hartwell Center. Amplicon sequence variants were classified into phylotypes using MaLiAmPi [21] based on their placement on a phylogenetic reference tree from full-length 16S rRNA alleles from the Ribosomal Database Project (RDP) [22]. All sequence data have been deposited in the National Center for Biotechnology Short Read Archive under the accession number PRJNA1056045.

### Microbiome analysis

Diversity, which depicts the evenness and richness of bacterial taxons within a community, was measured as the Balanced Weighted Phylogenetic Diversity (BWPD) [23]. Microbiome compositional attributes tested were ratios of phylotypes hypothesized to have equivalent roles in variable microbiomes and are normalized for sequence depth. Four specific ratios were constructed of screened phylotypes that differ between groups in abundance and variation identified by Corncob (release 0.2.0) [24] with a false discovery rate (FDR) of 0.01, as described previously [25]. Ratios screened are available in S2 Table.

### Statistical analysis

Statistical analyses were conducted using R version 4.3.1. Continuous and categorical variables were compared between groups using Wilcoxon-Mann-Whitney and Fisher’s exact tests. Logistic regression analyses were performed to identify whether the carriage of MRSA or MRCoNS was a risk factor for infections or infective symptoms and to identify if microbiome composition was associated with carriage of these microbes. Multivariate analysis controlling age, sex, race, and antibiotic use was considered for the association with infections and infective symptoms. For microbiome composition, multivariate analysis included adjustment for age, sex, race, and whether post-operative observation or concern for infection grouping.

## Results

### Prevalence of methicillin-resistant Staphylococcus

Nasal MRSA colonization was found in 6.5% patients admitted to the pediatric intensive care unit from 2021–2021, lower than the 27–45% observed from 2000–2002 ^26^. Despite the low MRSA prevalence, screening for MRCoNS revealed a 35% prevalence of nasal *mecA*+ colonization (MRSA and MRCoNS combined). Carriage of methicillin-resistant *Staphylococcus* did not differ between patients admitted to PICU for suspicion of infection and those admitted for post-operative observation (Table 1). In univariate analysis, patients with methicillin-resistant *Staphylococcus* were similar to those without in terms of age, sex, race, viral coinfection, asthma and antibiotic use. However, multivariate analysis showed carriage of methicillin-resistant *Staphylococcus* was independently associated with fever during PICU stay, with an adjusted odds ratio of 7.84 (95% confidence interval 1.95–31.45; p value of 0.004).

**Table 1:** Characteristics of patients admitted to LBCH PICU (Memphis, TN) and results of univariate analysis for risk factors for nasal colonization with either methicillin-resistant *Staphylococcus aureus* (MRSA) or methicillin-resistant coagulase-negative *Staphylococci* (MRCoNS).

	No Methicillin Resistant *Staphylococcus* Carriage (n = 40)	Carriage of Methicillin Resistant *Staphylococcus* (n = 22)	P value
**PICU Admission**			0.109
• Infection suspected • Post-operative observation	20 (50%) 20 (50%)	16 (72.7%) 6 (27.3%)	
**Age (median [range])**	1.7 [0.2–17] yr	1.6 [0.1–16.9] yr	0.848
**Sex (% female)**	19 (48.7%)	8 (36.4%)	0.426
**Race**			0.859
• Asian • African American • White	1 (2.5%) 15 (37.5%) 24 (60%)	0 7 (31.8%) 15 (68.2%)	
**Viral Coinfection**			>0.999
• Rhinovirus • RSV^^^	2 (5%) 7 (17.5%)	1 (4.5%) 3 (13.6%)	
**History of Asthma**	1 (2.5%)	2 (9.1%)	0.285
**Central Line**	7 (17.5%)	2 (9.1%)	0.368
**Antibiotics Prior to PICU Admission**	24 (61.5%)	16 (72.7%)	0.416
**Signs and symptoms during PICU stay:**			
• Fever • Hypothermia • Tachycardia • Hypotension • Tachypnea • Increased oxygen support • Elevated WBC • Elevated CRP	6 (15%) 2 (5%) 11(27.5%) 3 (7.5%) 12 (30%) 13 (32.5%) 4 (10%) 5 (12.5%)	11 (50%) 2 (9.1%) 9 (40.9%) 3 (13.6%) 9 (40.9%) 3 (13.6%) 3 (13.6%) 3 (13.6%)	0.006 0.610 0.395 0.657 0.413 0.136 0.691 >0.999

^^^Includes RSV + other viral coinfections

### Nasal microbiome traits in colonized critically ill children

**Fig 1:**
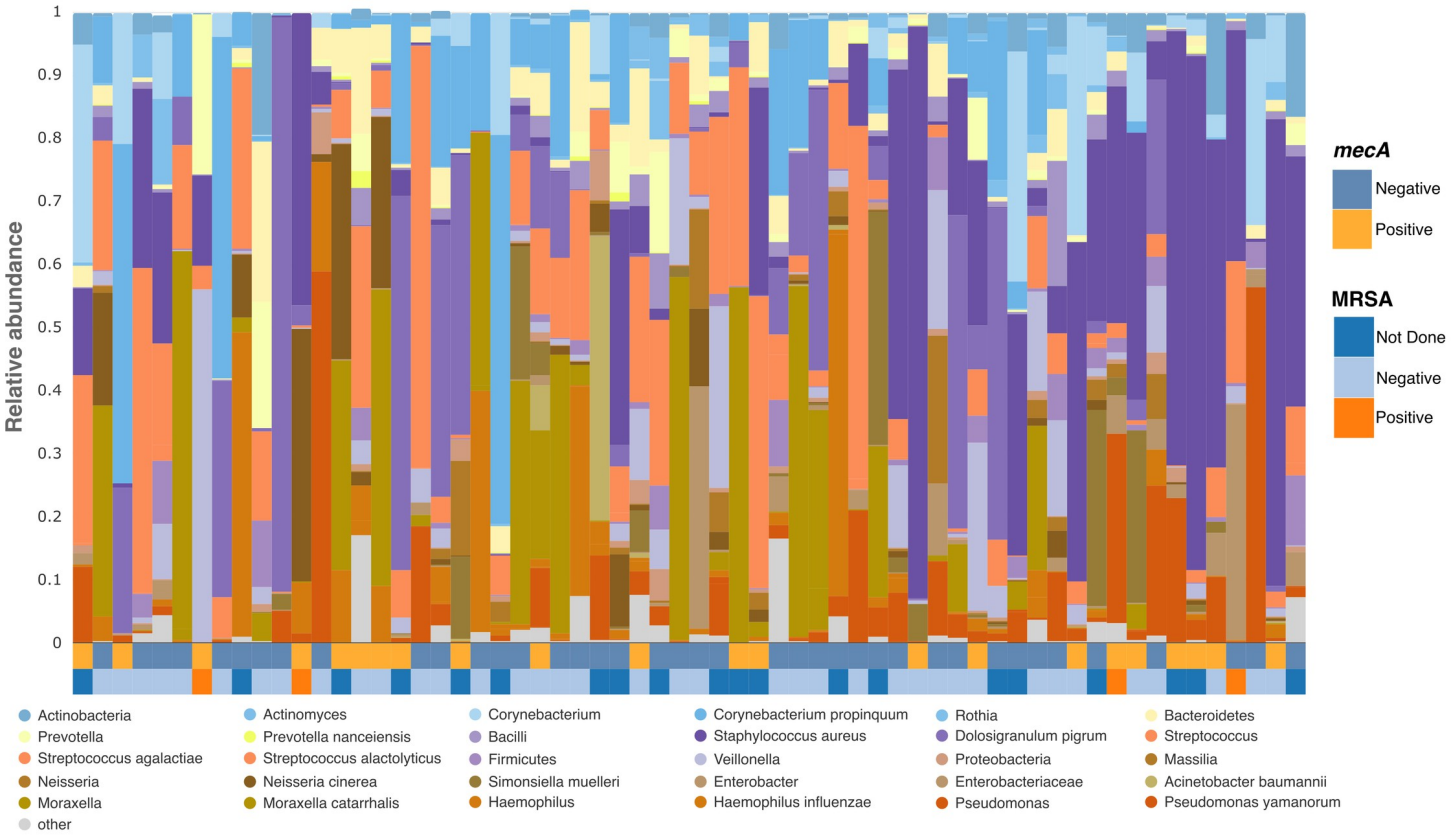
Nasal microbiota diversity. Diverse nasal microbiota on in 62 critically ill children with each microbiome a bar displayed as relative abundance; colors correspond to particular species or if not specified as other. Participants are ordered by age with heatmaps for methicillin-resistant *Staphylococcus* detection (*mecA*+, MRSA culture swab).

We characterized the nasal microbiota in critically ill children by analyzing admission nasal swabs using 16S ribosomal RNA gene sequencing. The composition of the nasal microbiota varied widely between individuals (Fig 1). Surprisingly, there was no link between low diversity of nasal microbiome and carriage of MRSA or MRCoNS (S1 Table). To identify unique taxa that influence colonization with methicillin-resistant *Staphylococcus*, we screened phylotypes that varied in distribution and abundance across multiple individuals [24] using a stringent criterion (FDR 0.01). These phylotypes selected to form ratios may originate from similar ecological niches within their communities.

After accounting for age, sex, race, and reason for PICU admission, we found that the ratio of anterior nasal mucosal microbes (*Streptococcus agalactiae*, *Gleimia europaea* and *Moraxella* sp.) or (*Corynebacterium propinquum* and *Massilia consociata*) to microbes more typical of deeper nasal mucosal sites (*Haemophilus haemolyticus*, *Prevotella nanceiensis*, and *Streptococcus alactolyticus*) could predict carriage with methicillin-resistant *Staphylococcus* (Table 2). The majority of identified nasal bacteria, including common commensals like *Dolosigranulum pigrum* and *Neisseria cinerea* and *Pseudomonas sp*., showed no significant association with methicillin-resistant *Staphylococcus* carriage (supplemental materials).

**Table 2:** Ratios of bacteria species found in the nasal microbiota of pediatric critically ill patients predicted carriage of methicillin-resistant *Staphylococcus*. Ratios were transformed using log_10_ before single and multivariate analysis to reduce skewness.

Ratio	Components	Unadjusted		Adjusted for age, admission reason, sex, race	
		Odds Ratio [95% CI]	P Value	Odds Ratio [95% CI]	P value
**1**	*S*. *agalactiae+ G*. *europaea + Moraxella sp*.	0.68 [0.49–0.95]	0.024	0.65 [0.45–0.94]	0.023
	*H*. *haemolyticus + P*. *nanceiensis + S*. *alactolyticus*				
**2**	*C*. *propinquum + M*. *consociata*	0.66 [0.50–0.86]	0.002	0.64 [0.47–0.88]	0.006
	*H*. *haemolyticus + P*. *nanceiensis + S*. *alactolyticus*				

## Discussion

The primary aim of PICU screening is to mitigate transmission of methicillin-resistant *Staphylococcus* species, including MRSA and CoNS, which are challenging to treat in critical care units. We found that MRSA prevalence among PICU admits was 6.5%, lower than expected in a highly endemic community. However, when the overall prevalence for methicillin-resistant *Staphylococcus* (MRSA and CoNS) colonization was 35%, which may be sufficient to facilitate transmission. We hypothesized that the nasal microbiome could identify individuals at elevated risk of colonization who have insufficient colonization resistance to these *Staphylococci*. We discovered microbiome signatures predicting methicillin-resistant *Staphylococcus* colonization within this cohort, which differed from prior literature suggesting no predictive nasal microbiota patterns [26]. The predictive ratio included several notable species, such as Group B Streptococcus (*S*. *agalactiae*) which in most children is commensal but carries significant clinical implications due to antimicrobial resistance potential. *Corynebacterium propinquum*, typically a benign colonizer of anterior nares, can participate in bacterial interspecies interactions through production of bacteriocins. *Moraxella* species, while generally commensal, may enhance susceptibility to viral infections and can harbor beta-lactam resistance genes. The deeper nasal species identified, including *Haemophilus haemolyticus* and *Prevotella nanceiensis*, are typically non-pathogenic anaerobes that may contribute to colonization resistance through niche competition and metabolic interactions. These species roles in the nasal ecosystem could inform future strategies for preventing methicillin-resistant *Staphylococcus* colonization.

This study has several limitations. The sample size was restricted due to changes in PICU census and sample availability during the COVID-19 pandemic. Excluding patients with COVID-19 also reduced the number of eligible patients. Expecting higher MRSA prevalence, we combined colonization from MRSA and CoNS, acknowledging potential differences in colonization behaviors and risk factors in these genetically distinct species. Prophylactic antibiotics in post-operative patients and treatment antibiotics in patients with suspected infections could have altered their microbiomes. While this study focused on broad microbiome characterization, future work should include both antimicrobial susceptibility testing and multilocus sequence typing (MLST) of isolates. Susceptibility testing to commonly used PICU antibiotics would guide treatment decisions, while sequence typing could reveal whether particular Staphylococcus lineages associate with distinct microbial communities. Together, these approaches would enhance our understanding of methicillin-resistant Staphylococcus and improve prediction of colonization patterns in critically ill children.

Larger studies are needed to better understand methicillin-resistant *Staphylococcus* transmission in PICUs. Nonetheless, this study highlights the need to consider methicillin-resistant CoNS in screenings and highlights that risk is not uniform across PICU patients (as some may have colonization resistance from their nasal flora). This study was not sufficiently powered to determine if nasal colonization was associated with other outcomes, but it is intriguing that fever was significantly linked with methicillin-resistant *Staphylococci* colonization. Further evidence from larger studies would be influential in understanding what interventions are beneficial in the PICU setting to limit transmission.

## Supporting information

S1 Fig Concordance qPCR/culture. (PDF)

S2 Fig Diversity. (PDF)

S1 Table Phylotype ratios included. (PDF)

S2 Table Infective symptoms association with methicillin-resistant *Staphylococcus* carriage. (PDF)

S3 Table Microbiome predictors of methicillin-resistant *Staphylococcus* carriage. (PDF)
