# Supplementary material for: Nasal microbiota predictors for methicillin resistant Staphylococcus colonization in critically ill children
Source: PLoS One. 2025 Jan 15;20(1):e0316460. doi: 10.1371/journal.pone.0316460 (PMC11734933; doi:10.1371/journal.pone.0316460)
Supplement: S1 Fig — (PDF) [file pone.0316460.s001.pdf]

1) Supplementary Methods: Characterization of methicillin resistant Staphylococcus

For each participant, samples were collected for microbial testing (both microbiome composition and mecA presence was determined from same DNA extraction). Presence of methicillin resistant Staphylococcus was based on PCR as quantitative PCR using the primers and probes for both the mecA and orfx-SCC junction as described in Kim et al [1] and for presence of staphylococcus we used a quantitative PCR using the primers and probes for the tuf genes as described in Martineau et al[2].

To validate this identification method for methicillin resistant Staphylococcus, we checked concordance to culture results (Figure S1). We found that a single individual that was culture negative but the qPCR suggested the presence of MRSA (mecA+ and orfX+). Two individuals were culture positive but the qPCR did not suggest the presence of MRSA (mecA+ and orfX-). The sensitivities of these assays are different and the samples coming from different nostrils may have contributed to differences in sampling collection to explain these discordances. Note that 20 individuals did not have their MRSA screening swab sent for culture.

	mecA		***mecA+orfX***	
**MRSA Screening**	**Negative**	**Positive**	**Negative**	**Positive**
**Negative**	27	11	37	1
**Positive**	1	3	2	2
**Not Done**	12	8	18	2

Figure S1: Individuals had separate swabs taken of opposing nostrils for culture and microbial detection of mecA via qPCR. The mecA gene can be present in both S. aureus and coagulase negative Staphylococcus. The first column is for whether the mecA qPCR was positive or negative. The second column corresponds to whether both mecA qPCR and orfX qPCR were positive which should only recognize MRSA.

References

[1] Jeong-Uk Kim, Choong-Hwan Cha, Hae-Kyong An, Ho-Jun Lee, and Mi-Na Kim. Multiplex Real-Time PCR Assay for Detection of Methicillin-Resistant Staphylococcus aureus (MRSA) Strains Suitable in Regions of High MRSA Endemicity. *Journal of Clinical Microbiology*, 51(3):1008–1013, March 2013. Publisher: American Society for Microbiology.

[2] F. Martineau, F. J. Picard, D. Ke, S. Paradis, P. H. Roy, M. Ouellette, and M. G. Bergeron. Development of a PCR assay for identification of staphylococci at genus and species levels. *Journal of Clinical Microbiology*, 39(7):2541–2547, July 2001.
