# Supplementary material for: Nasal microbiota predictors for methicillin resistant Staphylococcus colonization in critically ill children
Source: PLoS One. 2025 Jan 15;20(1):e0316460. doi: 10.1371/journal.pone.0316460 (PMC11734933; doi:10.1371/journal.pone.0316460)
Supplement: S2 Fig — (PDF) [file pone.0316460.s002.pdf]

Supplementary Materials 2: Microbiome Diversity and Methicillin-resistant Staphylococci colonization

Diversity is a measure of both the evenness and richness of bacterial taxons within a community. Phylogenetic diversity is less prone to error from over–classification, has grounding in classical phylogenetic diversity (improves interpretation of whether differences are biologically relevant/significant) and provides more discrimination than OTU methods or sequence variation richness. Given that taxonomies have inconsistent phylogenetic relationships in bacteria we have elected to use Balanced Weighted Phylogenetic Diversity (BWPD) to measure diversity in our samples[1]. We found that phylogenetic diversity did not differ significantly between those colonized with methicillin resistant staphylococcus and uncolonized. (Figure S2).

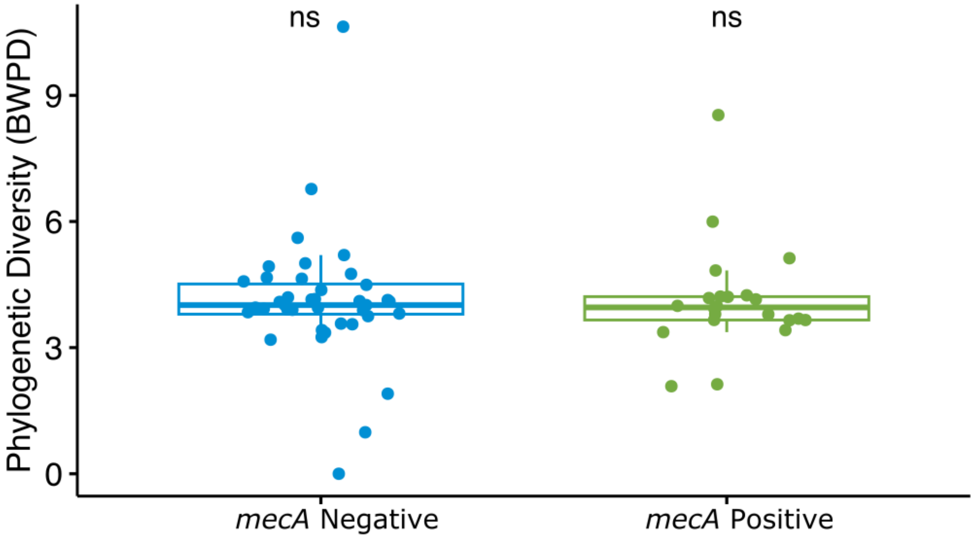

Figure S2: Phylogenetic Diversity (BWPD) of nasal microbiota in critically ill children who were colonized with methicillin resistant Staphylococcus (mecA positive) and those who were not (mecA negative). Not statistically significant difference between groups as determined by Kruskal-Wallis.

References

[1] Connor O. McCoy and Frederick A. Matsen. Abundance-weighted phylogenetic diversity measures distinguish microbial community states and are robust to sampling depth. *PeerJ*, 1:e157, sep 2013.
