# Supplementary material for: Nasal microbiota predictors for methicillin resistant Staphylococcus colonization in critically ill children
Source: PLoS One. 2025 Jan 15;20(1):e0316460. doi: 10.1371/journal.pone.0316460 (PMC11734933; doi:10.1371/journal.pone.0316460)
Supplement: S1 Table — (PDF) [file pone.0316460.s003.pdf]

Supplementary Materials 3: Composition Attributes and Phylotype Ratios

As composition can vary widely between individuals within a group and important taxa may be absent in various communities, we screened for phylotypes that persistently vary in distribution and abundance between those colonized with methicillin resistant Staphylococcus and those who were not as identified by Corncob release 0.2.0 (Count Regression for Correlated Observations with the Beta binomial)[1] with a false discovery rate of 0.01. Screened phylotypes were included in preliminary and final composition attribute ratios if variance persisted when outliers excluded (modified mean). Composition ratios of absolute abundances were constructed from screened phylotypes, with excess zeros of phylotype abundances handled with small positive constant (1) before performing read count ratio and log transformations similar in approach to ANCOM[2]. Ratios that resulted in describing similar taxonomies were discarded in favor of simpler ratios that had fewer total phylotypes. The following (Table S1) is a complete list of the ratios that were considered in this study (all ratios were tested against all outcomes).

Ratio	Phylotypes (if applicable)	Taxon Represented
ratio 1	pt___00136 + pt___00162 + pt___00193 + pt___00004	Streptococcus agalactiae + Gleimia europaea + Moraxella
	pt___00068 + pt___00030 + pt___00118	Haemophilus haemolyticus + Prevotella nanceiensis + Streptococcus alactolyticus
ratio 2	pt___00007 + pt___00091	Corynebacterium propinquum + Massilia consociata
	pt___00068 + pt___00030 + pt___00118	Haemophilus haemolyticus + Prevotella nanceiensis + Streptococcus alactolyticus

Table S1: Phylotype ratios include compositional attributes that were either chosen as they differed in variance and abundance between groups.

References

[1] Bryan D. Martin, Daniela Witten, and Amy D. Willis. Modeling microbial abundances and dysbiosis with beta-binomial regression. *The Annals of Applied Statistics*, 14(1), mar 2020.

[2] Siddhartha Mandal, Will Van Treuren, Richard A. White, Merete Eggesbø, Rob Knight, and Shyamal D. Peddada. Analysis of composition of microbiomes: a novel method for studying microbial composition. *Microbial Ecology in Health & Disease*, 26(0), may 2015.
