# Supplementary material for: Nasal microbiota predictors for methicillin resistant Staphylococcus colonization in critically ill children
Source: PLoS One. 2025 Jan 15;20(1):e0316460. doi: 10.1371/journal.pone.0316460 (PMC11734933; doi:10.1371/journal.pone.0316460)
Supplement: S2 Table — (PDF) [file pone.0316460.s004.pdf]

Supplementary Materials 4: Carriage of Methicillin-resistant Staphylococci and infective symptoms

To determine the association between carriage of methicillin-resistant Staphylococci and infective symptoms a logistic regression analysis was done on 5 symptoms which occurred in more than 10 participants: fever, increased oxygen support, tachycardia, tachypnea and suspicion for viral infection:

Infective symptoms	MRSA or MR-CoNS carriage	Unadjusted			Adjusted for age, sex, and race			Adjusted for antibiotics, age, sex, and race		
		OR	95% CI	P	OR	95% CI	P	OR	95% CI	P
Fever	Yes	5.67	[1.70; 18.91]	0.0048	7.69	[1.95; 30.29]	0.0036	7.84	[1.95; 31.45]	0.0037
	No	Ref.			Ref.			Ref.		
Increased oxygen	Yes	0.33	[0.08; 1.31]	0.115	0.40	[0.09; 1.80]	0.231	0.39	[0.08; 1.82]	0.230
	No	Ref.			Ref			Ref.		
Tachycardia	Yes	1.83	[0.61; 5.47]	0.283	1.99	[0.63; 6.28]	0.240	2.03	[0.63; 6.57]	0.238
	No	Ref.			Ref.			Ref.		
Tachypnea	Yes	1.62	[0.55; 4.79]	0.387	1.98	[0.62; 6.36]	0.250	2.07	[0.62; 6.89]	0.236
	No	Ref.			Ref.			Ref.		
Suspicion of Virus	Yes	0.80	[0.26; 2.44]	0.694	1.01	[0.23; 4.39]	0.992	1.02	[0.23; 4.42]	0.984
	No	Ref.			Ref.			Ref.		

Table S2: Logistic regression analysis showed a significant association between carriage with methicillin-resistant Staphylococcus and the occurrence of fever but not for other symptoms. OR, odds ratio; CI, confidence interval; P, p-value
