# Supplementary material for: Nasal microbiota predictors for methicillin resistant Staphylococcus colonization in critically ill children
Source: PLoS One. 2025 Jan 15;20(1):e0316460. doi: 10.1371/journal.pone.0316460 (PMC11734933; doi:10.1371/journal.pone.0316460)
Supplement: S3 Table — (PDF) [file pone.0316460.s005.pdf]

Supplementary Materials 5: Cox Regression Analysis- Microbiome Diversity, Ratios and Methicillin resistant Staphylococcus Carriage

Predictor	Unadjusted			Adjusted for age, sex, race and concern for sepsis		
	OR	95% CI	P	OR	95% CI	P
Log10 ratio1	0.68	[0.49; 0.95]	0.024	0.65	[0.45; 0.93]	0.019
Log10 ratio2	0.66	[0.50; 0.86]	0.0021	0.65	[0.48; 0.87]	0.0046
Diversity (BWPD)	0.88	[0.58; 1.35]	0.564	0.97	[0.59; 1.58]	0.890

Table S3: Logistic regression analysis showed a significant association between carriage with methicillin-resistant Staphylococcus and microbiome composition ratios but not microbiome diversity. OR, odds ratio; CI, confidence interval; P, p-value
